# P-85. Impact of Source Control on Clinical Outcomes in Prosthetic Joint Infections Caused by Staphylococcus lugdunensis

**DOI:** 10.1093/ofid/ofaf695.314

**Published:** 2026-01-11

**Authors:** Grethel N Hernandez, Wilmer Salazar, Aline Arif, Mohammad Alam, Paulette Pinargote

**Affiliations:** Louisiana State University Health Sciences Center Shreveport, shreveport, LA; Universidad Catolica Santiago de Guayaquil, Shreveport, Louisiana; LSU Health Shreveport, Shreveport, Louisiana; Louisiana State University Health Sciences Center, Shreveport, LA, USA, shreveport, Louisiana; Louisiana State University Health Sciences Center Shreveport, shreveport, LA

## Abstract

**Background:**

Staphylococcus lugdunensis is an uncommon but clinically significant pathogen increasingly implicated in prosthetic joint infections (PJI). It displays a pathogenic profile similar to Staphylococcus aureus, with aggressive soft tissue invasion and deep-seated infection. This study aimed to evaluate the association of source control, antibiotics resistance with clinical and microbiologic outcomes in patients with S. lugdunensis PJI.

**Methods:**

We conducted a retrospective cohort study of 47 patients with S. lugdunensis PJI treated at Ochsner facilities from 2012 to 2024. Patients were categorized based on whether they received surgical source control. Outcomes included death, symptom persistence or recurrence, microbiologic cure, repeat surgery, and use of chronic oral suppressive therapy. Bivariate analysis and multivariable logistic regression were used. Subgroup analysis evaluated antibiotic class (β-lactam vs. glyco-/lipopeptides) and resistance patterns.

**Results:**

Among 47 patients with Staphylococcus lugdunensis PJI (29 with hardware removal, 18 retained hardware), source control was linked to better outcomes: 84.2% without source control had persistent symptoms vs. 39.3% with source control (p = 0.003; OR 8.24). No significant differences were seen in infection recurrence (p = 1.0), repeat surgery (p = 0.32), symptom relapse (p = 0.64), or need for chronic suppressive antibiotics (p = 0.73). Demographics were similar between groups. Additionally, treatment with beta-lactams and presence of oxacillin resistance were not independently associated with clinical or microbiologic outcomes.

**Conclusion:**

Hardware removal was associated with improved clinical cure. While not all findings reached statistical significance, the overall trend supports source control as a key factor in recovery. Antibiotic choice did not appear to influence outcomes, though prospective studies are warranted.

**Disclosures:**

All Authors: No reported disclosures

